# Chromosome alterations in human hepatocellular carcinomas correlate with aetiology and histological grade – results of an explorative CGH meta-analysis

**DOI:** 10.1038/sj.bjc.6602448

**Published:** 2005-03-01

**Authors:** P Moinzadeh, K Breuhahn, H Stützer, P Schirmacher

**Affiliations:** 1 Institute of Pathology, Joseph-Stelzmann Str. 9, University of Cologne, 50931 Cologne, Germany; 2 Institute of Medical Statistics, Informations and Epidemiology, University of Cologne, 50931 Cologne, Germany; 3 Center for Molecular Medicine, University of Cologne, Joseph-Stelzmann Str. 9, 50931 Cologne, Germany

**Keywords:** hepatocellular carcinoma, hepatocarcinogenesis, hepatitis-B virus, hepatitis-C virus, comparative genomic hybridisation, meta-analysis

## Abstract

All available comparative genomic hybridisation (CGH) analyses (*n*=31, until 12/2003) of human hepatocellular carcinomas (HCCs; *n*=785) and premalignant dysplastic nodules (DNs; *n*=30) were compiled and correlated with clinical and histological parameters. The most prominent amplifications of genomic material were present in 1q (57.1%), 8q (46.6%), 6p (22.3%), and 17q (22.2%), while losses were most prevalent in 8p (38%), 16q (35.9%), 4q (34.3%), 17p (32.1%), and 13q (26.2%). Deletions of 4q, 16q, 13q, and 8p positively correlated with hepatitis B virus aetiology, while losses of 8p were more frequently found in hepatitis C virus-negative cases. In poorly differentiated HCCs, 13q and 4q were significantly under-represented. Moreover, gains of 1q were positively correlated with the occurrence of all other high-frequency alterations in HCCs. In DNs, amplifications were most frequently present in 1q and 8q, while deletions occurred in 8p, 17p, 5p, 13q, 14q, and 16q. In conclusion, aetiology and dedifferentiation correlate with specific genomic alterations in human HCCs. Gains of 1q appear to be rather early events that may predispose to further chromosomal abnormalities. Thus, explorative CGH meta-analysis generates novel and testable hypotheses regarding the cause and functional significance of genomic alterations in human HCCs.

Hepatocellular carcinoma (HCC) is one of the most prevalent cancers with steadily increasing incidence even in developed countries, such as Europe and the United States (El-Serag, 2002). It is believed that human hepatocarcinogenesis persists several decades normally starting from chronic liver disease over cirrhosis, premalignant precursor lesions (dysplastic nodules (DNs), ‘early’ HCC upto fully malignant HCC with angioinvasive and metastatic potential (reviewed in Kern *et al*, 2002a). In more than 80% of the cases, a well-defined aetiology (e.g. viral infection, aflatoxin B1 exposure, and chronic alcohol abuse) is associated with the development of HCC. Hepatitis B virus (HBV) infection is thought to contribute by two different mechanisms to hepatocarcinogenesis: chromosomal integrations of viral DNA with destabilising effects for the host genome (Luber *et al*, 1996) and expression of viral transactivating factors (HBxAg and preS/SAg; Kekule *et al*, 1990; Koike, 1995). The oncogenic potential of hepatitis C virus (HCV) infection has been linked to the viral transcriptional activator NS5A (Kato *et al*, 1997) and also to the core polypeptide (Moriya *et al*, 1998). Moreover, tumorigenic properties of aflatoxin B1 are linked to somatic G/T transversion in codon 249 of *TP53* (Ozturk, 1999). Furthermore, several cellular factors have been implicated in the pathogenesis of HCC (e.g., *TP53* (p53; Feitelson, 1998), *CTNNB1* (*β*-catenin; Prange *et al*, 2003), *RB1* (retinoblastoma; Zhang *et al*, 1994), *COX2* (cyclooxygenase-2; Kern *et al*, 2002b), *IGF2* (insulin-like growth factor-II; Breuhahn *et al*, 2004), and *CDH1* (E-cadherin; Matsumura *et al*, 2001)).

Studies of the overall chromosomal alterations in carcinomas are based on loss of heterozygosity (LOH) analyses and comparative genomic hybridisation (CGH). Comparative genomic hybridization is a fluorescence-based technique that is used for the detection of chromosomal imbalances in tissues or cell populations. Areas representing loss of genomic material may contain tumour suppressor genes, while gains may harbour dominant protumorigenic factors (e.g. oncogenes and growth factors) relevant for respective tumour entity. The power of CGH lies in its potential for a more or less unbiased whole genome screening; the main limitation is its relatively low resolution. By CGH several chromosomal regions carrying tumour-relevant genes (e.g. oncogenes) have been identified in solid tumours and haematological malignancies (reviewed in Weiss *et al*, 2002).

However, many information regarding relevant chromosomal rearrangements that may play a central role in the development of HCC are still missing. Meanwhile, over 30 different in part small CGH studies have generated a wealth of over 700 analysed HCCs that await comprehensive and comprising interpretation. The aim of this study was to compile the information from all available CGH data of human DNs/HCCs and to correlate these alterations with aetiology and histological grading. The results demonstrate a significant association of specific genomic imbalances with viral aetiology and histological grade, and generate further testable hypotheses.

## MATERIALS AND METHODS

### Comparative genomic hybridisation-studies

Overall, 785 different CGH analyses of HCCs are available from 31 studies (public NCBI database PubMed; published until 12/2003; see Supplementary online material). Inclusion criteria for the CGH studies were complete data profiles and explicit tumour classification (HCC, DN) as well as similar software and labelling systems and used thresholds (0.7–0.8 and 1.2–1.3). Information regarding the HBV status was present in 428 cases (244 HBV-positive and 184 HBV-negative cases). The HCV status was available in 338 cases (110 HCV-positive and 228 HCV-negative cases).

Tumour grading was given in 199 cases (126 cases with low grade (G1/2) and 73 cases with high-grade (G3/4) HCCs). Overall 30 CGH analyses of DNs were collected from four different studies (see Supplementary online material).

### Data recording and statistical analyses

All CGH data were recorded in a standardised fashion: each chromosome arm was divided from 0 to 100 (centromere to telomere direction) and gains, losses, or normal ratio were evaluated in steps of five. From these data, the respective charts and ideograms were displayed. Moreover, the data were submitted to statistical analyses using SPSS. However, when only parts of a chromosomal arm were affected, it was scored as a complete loss of the arm in the summarising tables. When specific chromosomal aberrations occurred in 20% of all analysed cases, they were rated to be high frequency. These aberrations were specifically submitted to further examination. Statistical evaluation was performed using explorative *χ*^2^ test. *P*-values cited were not corrected for multiple testing. The list of known tumour relevant genes localised in the chromosomal regions with imbalances of highest frequency was determined using the OMIN database (Online Mendelian Inheritance of Man).

## RESULTS

### Predominant chromosomal alterations in human HCCs

The complete meta-analysis of all available CGH data (*n*=785 HCCs) revealed that gains of chromosomal material were most prevalent in 1q (57.1% of the cases), 8q (46.6%), 6p (22.3%), and 17q (22.2%), and losses were most frequently present in 8p (38%), 16q (35.9%), 4q (34.3%), 17p (32.1%), and 13q (26.2%) (Table 1). These data testify amplifications and deletions of chromosomal arms on which oncogenes (e.g. *MYC* on 8q24) and tumour suppressor genes (e.g. *RB1* on 13q14) are located. Furthermore, several genes with known and potential protumorigenic functions (e.g. modulators of the WNT-signalling pathway like *FZD3*, *WISP1*, *SIAH-1*, and *AXIN2*) have been described to be grouped on respective chromosomal arms (Table 1).

### Hepatocellular carcinoma aetiology correlates with specific genomic imbalances

**Figure 1:**
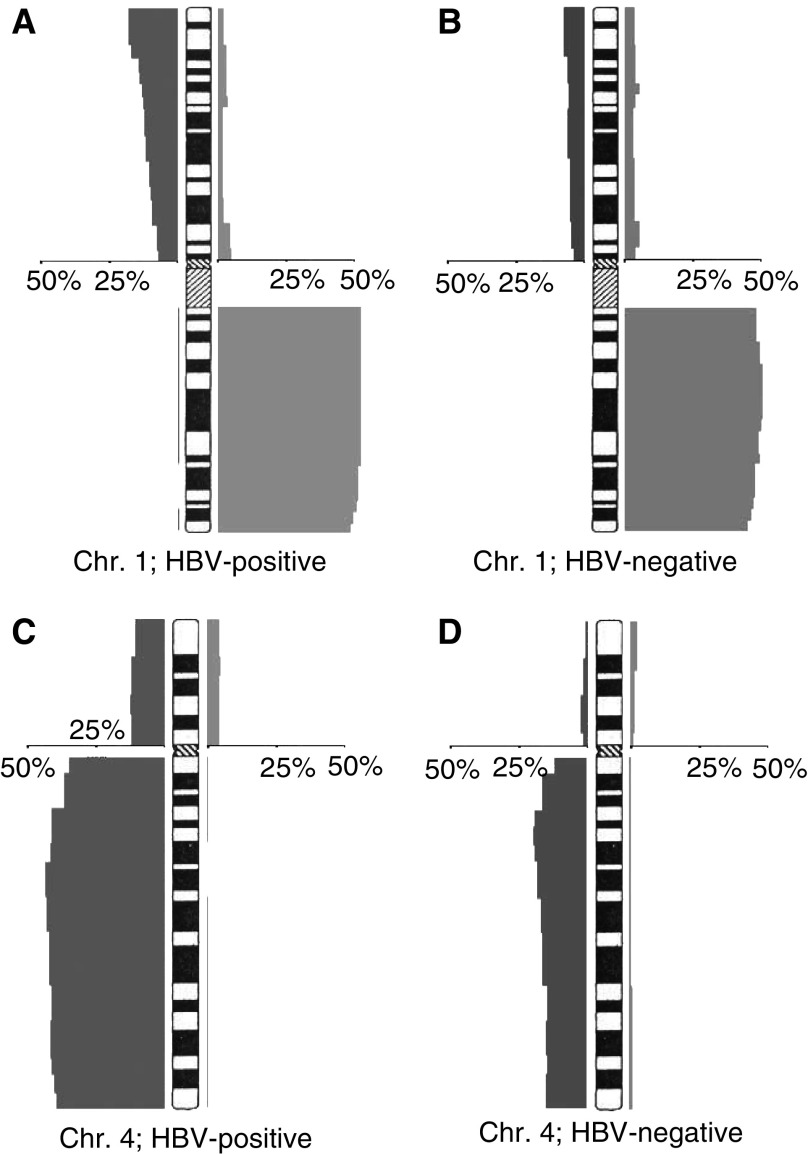
Distribution of genomic imbalances (*n*=428) with regard to HBV aetiology (for details, see Table 2). Data are exemplarily demonstrated for chromosomes 1 (no differences between (**A**) HBV-positive and (**B**) HBV-negative HCCs) and four (significant difference between (**C**) HBV-positive and (**D**) HBV-negative HCCs).

**Figure 2:**
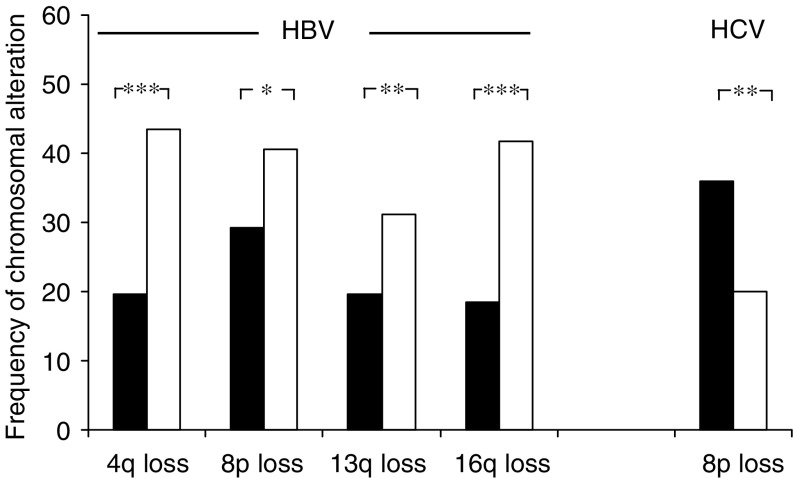
Graphical comparison of significant high-frequency genomic alterations in HBV- or HCV-negative (black bars) and HBV- or HCV-positive human HCCs (white bars). ^*^P<0.05; ^**^P<0.01; ^***^P<0.001.

In 428 CGH analyses of HCCs, the HBV status was given (244 HBV-positive and 184 HBV-negative cases). When HBV-positive and -negative cases were compared, losses at 4q (43.4 *vs* 19.6%), 16q (41.8 *vs* 18.5%), 13q (31.1 *vs* 19.6%), and 8p (40.6 *vs* 29.3%) were positively correlated with HBV aetiology (Figures 1 and 2; Table 2). The 338 HCCs with known HCV status consisted of 110 HCV-positive and 228 HCV-negative cases. Among these cases, only losses of 8p were more frequent in HCV-negative cases (36 *vs* 20%) (Figure 2; Table 2). No other significant association of genomic imbalances in HCCs with viral infections was found (data not shown).

### Histological grade of HCCs correlates with genomic imbalances

Next we analysed whether the histological grade (tumour differentiation) of the HCCs correlated with the observed pattern of chromosomal aberrations (Table 3). In 199 cases the histological grade was specified (126 low-grade (G1/G2) and 73 high-grade (G3/G4) HCCs). Losses of 13q (43.8 *vs* 11.9%) and 4q (45.2 *vs* 23%) were significantly more frequent in high-grade HCCs while the other high-frequency genomic imbalances did not correlate with the tumour grade (data not shown).

### Correlations between different genomic imbalances

We next tested whether any of the high-frequency imbalances (⩾20%) were statistically connected. Altogether, 498 CGH analyses of HCCs given as individual profiles were accessible to this analysis. Deletions of genomic material on 4q, 13q, and 16q frequently coincided. Furthermore, gains of 1q were positively associated with all other high-frequency alterations, except gains on chromosome 17q (Table 4).

Hepatocellular carcinomas with gains on 1q (*n*=293; 58.8%) were compared to cases without genomic alterations of 1q (*n*=205; 41.2%) regarding the occurrence of additional chromosomal imbalances per case. A significantly higher number of chromosomal imbalances were found in HCCs with 1q gains (6.74 imbalances per case) than in HCCs without 1q gains (3.40 imbalances per case; *P*<0.05).

### Genomic macroimbalances in human DNs

**Figure 3:**
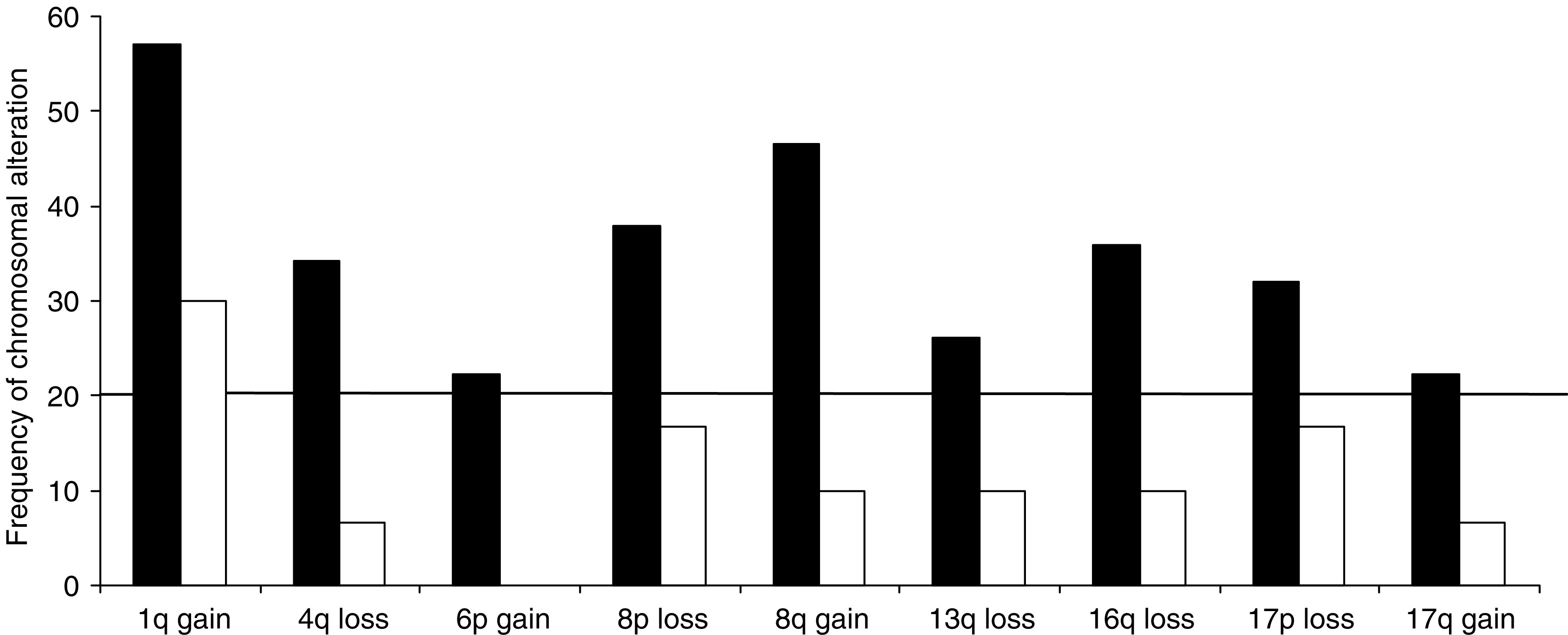
Direct comparison of chromosomal high-frequency alterations (⩾20%) in HCCs (black bars) and related DNs (white bars).

Four different studies have analysed a limited number of DNs (*n*=30) by CGH. Altogether, DNs showed genomic imbalances, although at a lower frequency as compared to HCCs. Gains were detectable in 1q (30%) and 8q (10%), while losses were most prevalent at 8p (16.7%), 17p (16.7%), 5p, 13q, 14q, and 16q (all 10%); (Figure 3). In DNs only gains on 1q were frequently detectable in more than 20% of all analysed cases.

## DISCUSSION

This meta-analysis of CGH data of human HCCs has unravelled several correlations between aetiology as well as histological grade and genomic imbalances. However, this analysis is afflicted with several restrictions as a result of not identical criteria in different studies.

- *Aetiology*: Some well-established reasons for human HCCs, such as chronic alcoholic liver disease, genetic haemochromatosis, and aflatoxin B1 exposure, are either ill defined and/or have not been evaluated in a sufficient number of cases studied by CGH. Therefore, we restricted our analysis to the potential associations with the HBV and HCV status. Analyses of the viral status carried some inconsistency by itself. Especially, in most studies HBV aetiology was evaluated by assaying for HBsAg in the serum. In contrast, HBV may exhibit its protumorigenic effects despite HBV seroconversion (Paterlini and Brechot, 1991). Significant correlations of chromosomal rearrangements with HBV aetiology have been unravelled, but in our analysis they may have been even more pronounced, if complete HBV serology would have been performed in all studies.
- *Lack of a uniform histological grading*: Several grading schemes are in use for HCC (Edmondson and Steiner, 1954), and in many studies it remains unclear which grading scheme has been applied. Due to this fact and in order to generate groups large enough for statistical analysis, it was necessary to combine grades 1 and 2 (well differentiated) and 3 and 4 (poorly differentiated), which is in accordance which numerous studies in other carcinomas.

Despite these restrictions, a number of high-frequency genomic imbalances have been clearly identified (Figure 1). With some of these aberrations the expression of tumour relevant genes may be correlated, such as *RB1* (13q), *CDH1* (16q), *SIAH1* (16q), and *TP53* (17p). Interestingly, some of these genes have been described to participate in pivotal signalling pathways frequently dysregulated in HCCs (reviewed in Ozturk, 1999). While the p53 protein is involved in different cellular processes (e.g. apoptosis, cell cycle, and differentiation; Levine, 1997), dysregulation of retinoblastoma protein (*RB1*), p21^WAF^ (*CDKN1A*), and CyclinA2 (*CCNA*) directly regulate initiation and progression of DNA synthesis (Zhang *et al*, 1994; Qin *et al*, 1998). Notably, several components of the WNT-signalling pathway were localised on aberrant genomic regions (*WNT14*, *LEF1*, *FZD3*, *WISP1*, *SIAH-1*, and *AXIN2*). This is of particular interest since upto 40% of all HCCs exhibit nuclear enrichment of *β*-catenin, the transcriptional activator of the WNT pathway (Cui *et al*, 2003; Prange *et al*, 2003). However, higher resolution techniques, such as matrix-CGH (Wessendorf *et al*, 2002) and expression analyses, will have to verify the expected expression changes for most genes.

Losses of 4q, 13q, 16q, and 8p were significantly correlated with HBV aetiology. Although the responsible genes are currently unknown, HBV-related chromosomal rearrangements have been mapped to 4q (Blanquet *et al*, 1988; Pasquinelli *et al*, 1988; Buetow *et al*, 1989). This suggests that in addition to well-established HBV-associated oncogenic mechanisms such as transcriptional transactivation by viral proteins (Wollersheim *et al*, 1988; Caselmann *et al*, 1990; Kekule *et al*, 1990) and nonhomologous chromosomal integration of viral DNA (Nagaya *et al*, 1987), specific corresponding host factors involving tumour suppressor genes positioned at the respective chromosomal loci may exist. Identification of these responsible genes may generate further insight into the mechanisms of HBV-induced oncogenesis.

Although the number of analysed premalignant lesions (DNs) is still low, several conclusions can already been drawn: Significant genomic imbalances can be detected in DNs and they partly resemble the changes present in HCCs, although at a lower frequency. This further supports the current hypothesis that DNs are indeed the immediate premalignant precursors of HCCs.

Another important question is the time point at which the different genomic imbalances occur during the process of hepatocarcinogenesis. Gains of 1q are the most frequent alteration in DNs and appear at equal frequencies in well and poorly differentiated HCCs; this suggests that amplifications of 1q represent an early protumorigenic change that mostly precedes malignant transformation. In contrast, deletions of 4q and 13q are found significantly more frequent in poorly differentiated HCCs. This suggests that both alterations are late progression events that typically occur after malignant transformation.

**Figure 4:**
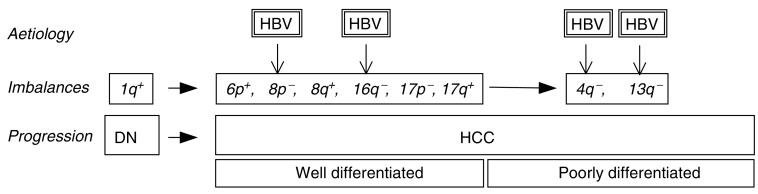
Schematic display of the CGH meta-analysis showing high-frequency chromosomal changes (*imbalances*) in association with aetiology and progression.

Statistical analyses demonstrate that 1q gains are positively correlated with all other high-frequency alterations, suggesting that they may predispose to chromosomal alterations. Thus, the status of 1q may distinguish between two different molecular pathways in hepatocarcinogenesis, of which cases with 1q gains are characterised by early-on acquired genomic imbalances (‘mutator phenotype’, Figure 4). This hypothesis is further supported by our finding that HCCs with 1q gains carry significantly more additional chromosomal alterations as compared to HCCs without 1q amplifications.

Taking these considerations into account, the following hypotheses can be formulated:

- Dysplastic nodules are premalignant precursor lesions that already carry fixed genomic alterations.
- Specific aetiologies, especially chronic HBV infection, may lead to characteristic host genomic alterations. Deletions on chromosome 4q, 13q, and 16q strongly correlate with HBV aetiology and tumour progression (4q and 13q), and may therefore contribute to the functional loss of tumour suppressors.
- Gains of 1q are the predominant early genomic alterations. They are aetiology-independent and may further predispose to other chromosomal imbalances (‘mutator phenotype’).

These hypotheses will have to be tested experimentally by several means. Firstly, the data basis in regard to DNs is still restricted and it is worthwhile to increase the number of CGH analyses of DNs. Secondly, the resolution of conventional CGH analysis to identify regions of interest is limited (approximately 3–10 Mbp). This gap may in part be closed by high-resolution techniques such as matrix-CGH (down to approximately 100 kbp (Wessendorf *et al*, 2002; Pestova *et al*, 2004). Identification and functional analyses of potential target genes may finally unravel the mechanisms that predispose to secondary chromosomal changes (e.g. 1q) or aetiology-specific alterations (e.g. 4q).

## Figures and Tables

**Table 1:** Frequencies of chromosomal alterations in human HCCs

	**p-arm**				**q-arm**			
**Chromosome**	**Loss (%)^a^**	**Gain (%)^a^**	**High. Fre.^b^**	**Genes^c^**	**Loss (%)^a^**	**Gain (%)^a^**	**High. fre.^b^**	**Genes^c^**
1	15.4	5.2	—	—	0.6	**57.1**	**q21.1–q44**	*WNT14, FASL*
2	1.4	7.1	—	—	2.9	8	—	—
3	3.9	5	—	—	1.9	8.8	—	—
4	10.6	6	—	—	**34.3**	1.7	**q21.1–q35**	*LEF1, CCNA*
5	1.7	13.6	—	—	7.8	11.1	—	—
6	1	**22.3**	—	*PIM1, CDKN1A*	15	7.9	—	—
7	0.9	15	—	—	3.1	16.8	—	—
8	**38**	4.6	**p21.1–p22**	*FZD3, PLK3*	1.9	**46.6**	**q22.1–q24.3**	*MYC, WISP1*
9	14	3.3	—	—	11.1	2.9	—	—
10	2.7	8.3	—	—	11.1	4.1	—	—
11	5.4	4.3	—	—	10.2	9.4	—	—
12	6.5	2.4	—	—	2.9	6.9	—	—
13	0	0	—	—	**26.2**	7.4	**q14.1–q22**	*RB1, BRCA3*
14	0	0	—	—	11.3	4.1	—	—
15	0	0	—	—	5.4	4.6	—	—
16	16.8	3.4	—	—	**35.9**	1.8	**q12.1–q24**	*SIAH1, CDH1*
17	**32.1**	2.9	**p13**	*p53, HIC1*	3.7	**22.2**	**q23–q25**	*AXIN2, TIMP2*
18	4.1	5.5	—	—	10.8	5	—	—
19	6.9	5	—	—	3.8	10.4	—	—
20	2	14.9	—	—	0.9	18.6	—	—
21	0	0	—	—	8.8	2.2	—	—
22	0	0	—	—	6.4	2.8	—	—
X	5	11.2	—	—	4.5	15	—	—
Y	5.1	2.3	—	—	5.6	2.3	—	—

a Frequencies ⩾20% are highlighted in bold.

b Regions with highest frequency of imbalances on the respective chromosomal arm are highlighted in bold.

c Examples of known tumour-relevant genes located on the respective chromosomal high-frequency region.

**Table 2:** Comparison of significant high-frequency genomic imbalances (⩾20%; see Table 1) in human HCCs with HBV- (*n*=428) and HCV-aetiology (*n*=338)

	**HBV** *			HCV*		
**Chromosome**	**Negative (*n***=**184)**		**Positive (*n***=**244)**	**Negative (*n***=**228)**		**Positive (*n***=**110)**
1q (gain; %)	50.5	*P*=0.625	53.3	53.5	*P*=0.201	45.5

4q (loss; %)	**19.6**	***P*<0.0005**	**43.4**	26.3	*P*=0.896	27.3

6p (gain; %)	21.2	*P*=0.488	24.2	24.1	*P*=0.121	16.4

8p (loss; %)	**29.3**	***P*=0.019**	**40.6**	**36.0**	***P*=0.004**	**20.0**

8q (gain; %)	37.0	*P*=0.05	46.7	42.1	*P*=0.194	34.5

13q (loss; %)	**19.6**	***P*=0.008**	**31.1**	28.5	*P*=0.363	23.6

16q (loss; %)	**18.5**	***P*<0.0005**	**41.8**	28.1	*P*=0.898	27.3

17p (loss; %)	25.0	*P*=0.108	32.4	24.1	*P*=0.19	30.9

17q (gain; %)	17.4	*P*=0.389	20.9	19.3	*P*=0.766	17.3

* Significant differences are highlighted in bold (*P*<0.05).

**Table 3:** Comparison of significant high-frequency genomic imbalances (⩾20%; see Table 1) in human HCCs with regard to tumour grade (*n*=199; low grade: G1 and G2 tumours; high grade: G3 and G4 tumours)

	**Tumour grade** *		
**Chromosome**	**Low (G1+G2)**		**High (G3+G4)**
1q (gain; %)	54.0	*P*=0.459	60.3

4q (loss; %)	**23.0**	***P*=0.001**	**45.2**

6p (gain; %)	19.8	*P*=1.0	19.2

8p (loss; %)	32.5	*P*=0.876	34.2

8q (gain; %)	39.7	*P*=0.299	47.9

13q (loss; %)	**11.9**	***P*<0.0001**	**43.8**

16q (loss; %)	23.8	*P*=0.053	37.0

17p (loss; %)	33.3	*P*=0.443	39.7

17q (gain; %)	19.8	*P*=0.224	27.4


* Significant differences are highlighted in bold (*P*<0.05).

**Table 4:** Correlation analysis of genomic aberrations in human HCCs (in ⩾20%; *n*=498)

**Chromosome**	**1q (gain)**	**4q (loss)**	**6p (gain)**	**8p (loss)**	**8q (gain)**	**13q (loss)**	**16q (loss)**	**17p (loss)**	**17q (gain)**
1q									
gain	—	43	30.6	45.7	52.6	30.2	38.1	36.8	—
Ø	—	19.8	11.1	25.6	30.4	16.4	23.7	21.3	—

4q									
Loss	73.4	—	—	51.6	—	42	53.2	41	29.8
Ø	41.9	—	—	28.7	—	13.9	19.4	23.9	15.5

6p									
Gain	76.9	—	—	52.1	—	—	—	—	—
Ø	45.9	—	—	32.6	—	—	—	—	—

8p									
Loss	66.3	46.6	33.7	—	—	—	48.3	41	—
Ø	46.6	26.3	17.2	—	—	—	23.1	24.4	—

8q									
Gain	65.5	—	—	—	—	—	—	—	—
Ø	43.5	—	—	—	—	—	—	—	—

13q									
Loss	68.7	57.3	—	—	—	—	52.7	—	—
Ø	48.2	25.3	—	—	—	—	24.8	—	—

16q									
Loss	65	56.4	—	55.2	—	41.1	—	42.9	—
Ø	47.8	22.4	—	28.7	—	16.4	—	24.2	—

17p									
Loss	66.9	44.8	—	51.3	—	—	46.8	—	—
Ø	48.8	28.5	—	31.1	—	—	25.6	—	—

17q									
Gain	—	55	—	—	—	—	—	—	—
Ø	—	28.2	—	—	—	—	—	—	—

Only significant differences between distinct chromosomal imbalances (first line) and the possibles status of all other high-frequency alterations (gain/loss *vs* no changes) are shown (*P*<0.05).
