# Supplementary material for: Chromosome alterations in human hepatocellular carcinomas correlate with aetiology and histological grade – results of an explorative CGH meta-analysis
Source: Br J Cancer. 2005 Mar 1;92(5):935–41. doi: 10.1038/sj.bjc.6602448 (PMC2361895; doi:10.1038/sj.bjc.6602448)
Supplement: Supplementary online material [file 92-6602448x1.doc]

| **Hepatocellular carcinomas** | | |
| --- | --- | --- |
|  | *Data source (Publications until 12/2003)* | *Analysed HCCs* |
| 1 | Marchio et al., Genes Chromosomes Cancer. 1997 Jan;18(1):59-65. | 50 |
| 2 | Kusano et al., Hepatology. 1999 Jun;29(6):1858-62. | 41 |
| 3 | Lin et al., Eur J Cancer. 1999 Apr;35(4):652-8. | 42 |
| 4 | Qin et al., Cancer Res. 1999 Nov 15;59(22):5662-5. | 10 |
| 5 | Sakakura et al., Br J Cancer. 1999 Aug;80(12):2034-9. | 26 |
| 6 | Wong et al., Am J Pathol. 1999 Jan;154(1):37-43. | 47 |
| 7 | Chen et al., Gastroenterology. 2000 Aug;119(2):431-40. | 31 |
| 8 | Guan et al., Genes Chromosomes Cancer. 2001 Jan;30(1):110. | 50 |
| 9 | Harada et al., Jpn J Cancer Res. 2000 Nov;91(11):1119-25. | 3 |
| 10 | Marchio et al., Oncogene. 2000 Aug 3;19(33):3733-8. | 44 |
| 11 | Tornillo et al., J Pathol. 2000 Nov;192(3):307-12. | 41 |
| 12 | Wilkens et al., J Pathol. 2000 Sep;192(1):43-51. | 3 |
| 13 | Wilkens et al., Am J Clin Pathol. 2000 Dec;114(6):867-74. | 12 |
| 14 | Wong et al., Clin Cancer Res. 2000 Oct;6(10):4000-9. | 83 |
| 15 | Zondervan et al., J Pathol. 2000 Oct;192(2):207-15. | 26 |
| 16 | Balsara et al., Genes Chromosomes Cancer. 2001 Mar;30(3):245-53. | 52 |
| 17 | Collonge-Rame et al., Cancer Genet Cytogenet. 2001 May;127(1):49-52. | 16 |
| 18 | Kitay-Cohen et al., Cancer Genet Cytogenet. 2001 Nov;131(1):60-4. | 4 |
| 19 | Koo et al., Cancer Genet Cytogenet. 2001 Oct 1;130(1):22-8. | 24 |
| 20 | Niketeghad et al., Br J Cancer. 2001 Sep 1;85(5):697-704. | 21 |
| 21 | Shiraishi et al., Oncology. 2001;60(2):151-61. | 31 |
| 22 | Takeo et al., Cancer Genet Cytogenet. 2001 Oct 15;130(2):127-32. | 20 |
| 23 | Wang et al., Genes Chromosomes Cancer. 2001 Jul;31(3):221-7. | 20 |
| 24 | Wilkens et al., J Pathol. 2001 Apr;193(4):476-82. | 6 |
| 25 | Wong et al., Am J Pathol. 2001 Aug;159(2):465-71. | 36 |
| 26 | Chang et al., Cancer Lett. 2002 Aug 28;182(2):193-202. | 22 |
| 27 | Cheung et al., Cancer Res. 2002 Aug 15;62(16):4711-21. | 6 |
| 28 | Pang et al., Genes Chromosomes Cancer. 2002 Feb;33(2):150-9. | 4 |
| 29 | Kellner et al., Am J Clin Pathol. 2003 Feb;119(2):265-71. | 1 |
| 30 | Ng et al., J Pathol. 2003 Mar;199(3):345-53. | 11 |
| 31 | van Dekken et al., Acta Histochem. 2003;105(1):29-41. | 2 |

| **Dysplastic Nodules** | | |
| --- | --- | --- |
|  | *Data source (Publications until 12/2003)* | *Analysed DNs* |
| 1 | Zondervan et al., J Pathol. 2000 Oct;192(2):207-15. | 12 |
| 2 | Tornillo et al., Lab Invest. 2002 May;82(5):547-53. | 10 |
| 3 | Schirmacher et al., Pathol Res Pract. 2002 April; 198(3):230. | 6 |
| 4 | van Dekken et al., Acta Histochem. 2003;105(1):29-41. | 2 |
